# Machine Learning-Guided Multi-Cohort Transcriptomic Profiling Identifies *SPON1* and *ALDH1A2* as Diagnostic and Prognostic Biomarkers Linked to the Immune Microenvironment in High-Grade Serous Carcinoma

**DOI:** 10.3390/ijms27146263

**Published:** 2026-07-14

**Authors:** Roozbeh Heidarzadeh-Pilehrood, Homa Azizimazreah, Habibah Abdul Hamid

**Affiliations:** 1 Department of Obstetrics & Gynecology, Faculty of Medicine and Health Sciences, Universiti Putra Malaysia, Serdang 43400, Malaysia; 2 Department of Public Health, University of Nevada, Las Vegas, NV 89119, USA

**Keywords:** high-grade serous carcinoma, ovarian cancer, transcriptomics, machine learning, Leave-One-Dataset-Out validation, prognostic biomarkers, *ALDH1A2*, *SPON1*, tumor microenvironment

## Abstract

Reliable biomarkers for high-grade serous carcinoma (HGSC) with prognostic and microenvironmental relevance remain limited. Here, we developed a machine learning–guided cross-cohort transcriptomic framework to identify stable biomarkers in HGSC. Three GEO cohorts comprising 68 samples (34 HGSC and 34 normal) and 21,355 genes were integrated, and five classifiers were benchmarked under strict Leave-One-Dataset-Out (LODO) validation. Differential expression and random-effects meta-analysis were used to support cross-cohort feature prioritization, and external validation was performed in TCGA-OV tumors (*n* = 427) versus GTEx normal ovaries (*n* = 88). This framework identified a robust 22-gene consensus panel with strong cross-cohort discrimination between HGSC and normal tissue. Among these, *ALDH1A2* and *SPON1* emerged as the only two genes consistently prioritized by all five models. Prognostic analysis showed opposite clinical associations, with higher *ALDH1A2* linked to poorer progression-free and overall survival and higher *SPON1* linked to better outcomes. Immune-module analysis further demonstrated that predicted HGSC probability was positively associated with T cell, cytotoxic/NK, Treg, checkpoint, and inflammatory programs, indicating an immune-active yet immunoregulatory microenvironment. Together, these findings define a reproducible 22-gene HGSC signature and highlight *ALDH1A2* and *SPON1* as robust diagnostic and prognostic biomarkers.

## 1. Introduction

Epithelial ovarian cancer (EOC) remains the most lethal gynecologic malignancy, with high-grade serous carcinoma (HGSC) accounting for approximately 75% of cases. The aggressive nature and profound biological heterogeneity of HGSC pose significant challenges for early detection and long-term disease control [1]. Despite the clinical utility of CA-125 in monitoring, its limitations in sensitivity and specificity—particularly across different histological subtypes—highlight the urgent need for more robust, generalizable molecular signatures [2,3]. HGSC is characterized by complex transcriptional dysregulation involving DNA damage response, genomic instability, and extracellular matrix remodeling, all of which are deeply intertwined with the tumor microenvironment (TME) [4]. Specifically, the immune landscape has emerged as a critical determinant of HGSC progression and clinical outcomes [5]. Consequently, identifying biomarkers that demonstrate both cross-cohort stability and immunological interpretability is essential for advancing precision diagnostics and risk stratification in HGSC.

While machine learning (ML) has revolutionized transcriptomic biomarker discovery, the gap between in-sample accuracy and true external generalizability remains a critical challenge [6]. High performance often stems from information leakage—such as global feature selection—or cohort-specific artifacts. To ensure clinical relevance, we adopted a rigorous Leave-One-Dataset-Out (LODO) validation framework, where entire cohorts remain untouched during all preprocessing and model-fitting phases. To balance stability with predictive power, we integrated complementary algorithms: Elastic Net for sparse feature selection under multicollinearity [7], Ensemble methods (Random Forest and Gradient Boosting) to capture non-linear biological structures [8], and Support Vector Machines for robust high-dimensional classification [9]. Model efficacy was evaluated using threshold-independent ROC/AUC metrics [10], prioritizing feature consistency across diverse classifiers to derive a stable, translationally viable molecular signature.

**Figure 1:**
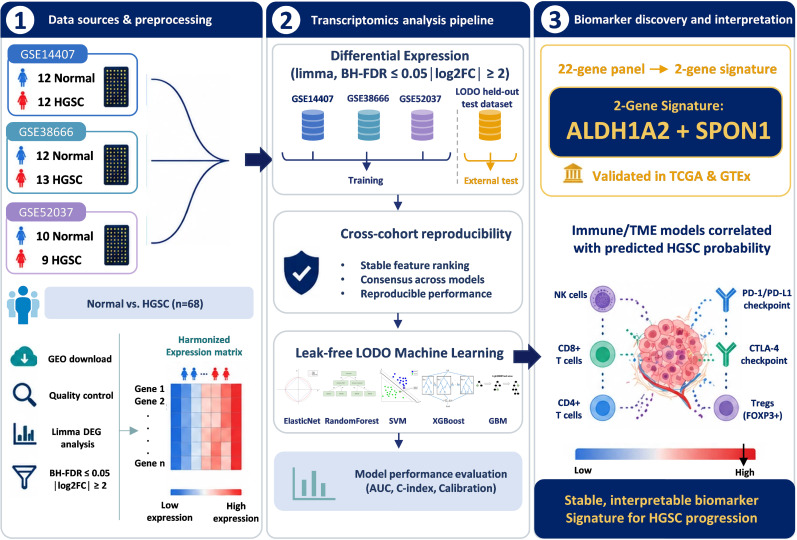
Graphical abstract of the cross-cohort machine learning and transcriptomic pipeline designed to identify a robust microenvironment-related signature in high-grade serous carcinoma.

In this study, we developed a robust, end-to-end framework prioritizing cross-cohort reproducibility and biological interpretability. By integrating independent GEO cohorts, our approach combines: (i) meta-analysis of differential expression with stringent FDR control; (ii) leakage-safe LODO validation to ensure model generalizability; and (iii) stability-based feature selection across diverse machine learning architectures to derive a refined diagnostic gene panel. Beyond classification, we contextualized these signatures through a systems-level immune scoring strategy, quantifying case–control shifts and their associated uncertainties using non-parametric inference and resampling-based confidence intervals. Our objective is to provide a highly stable, validated transcriptomic signature for HGSC risk stratification, alongside a fully transparent pipeline that integrates diagnostic precision with the underlying tumor-immune landscape (Figure 1).

## 2. Results

### 2.1. Cohorts’ Expression Matrices

**Figure 2:**
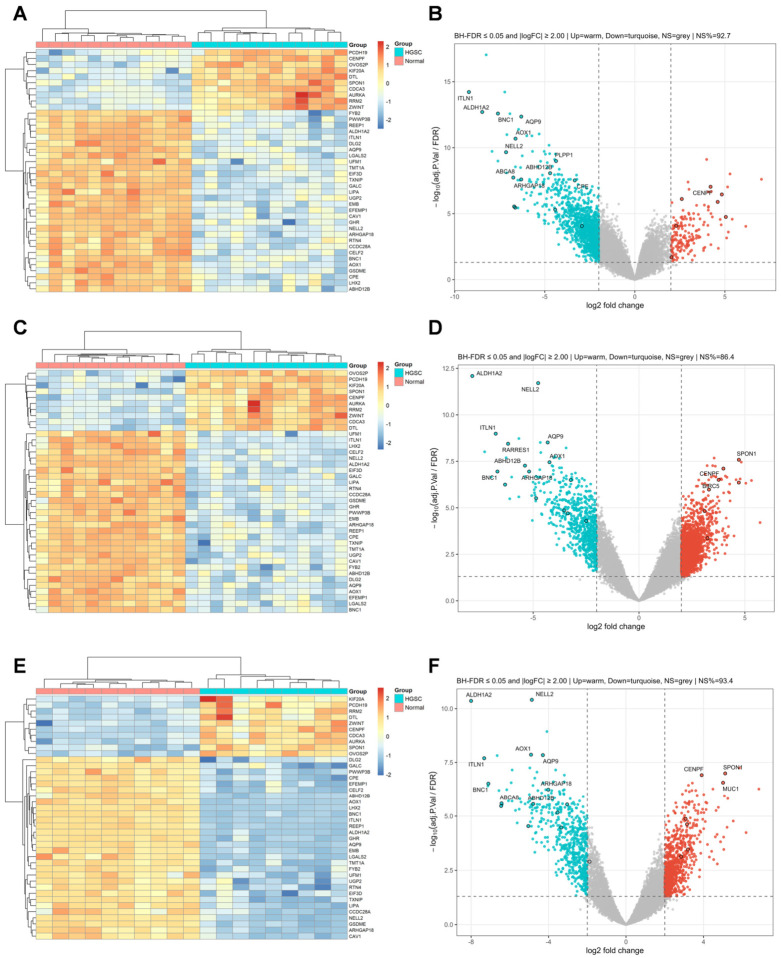
Cohort-specific differential expression landscapes (heatmaps and volcano plots). For each cohort, the top differentially expressed genes are visualized by heatmap (row-wise scaled expression) and volcano plot. Colors: Up-regulated in HGSC (warm/orange), Down-regulated in HGSC (turquoise), and non-significant genes (gray) when the ALL-genes limma table is used. Threshold lines indicate BH-FDR ≤ 0.05 and |log2FC| ≥ 2.0. (**A**,**B**) GSE14407: heatmap of the top-ranked genes and volcano plot of the cohort-wide limma results. (**C**,**D**) GSE38666: heatmap and volcano plot. (**E**,**F**) GSE52037: heatmap and volcano plot.

Figure S1, illustrates ML and cross-cohorts analysis pipeline in HGSC vs. Normal. We analyzed 68 samples across three independent GEO cohorts (GSE14407, GSE38666, and GSE52037). After rigorous preprocessing, a consensus transcriptome of up to 21,355 genes was established (Table S1). Differential expression analysis revealed highly consistent molecular signatures across cohorts, identifying 1411, 1812, and 1272 DEGs, respectively (BH-FDR ≤ 0.05, |log2FC| ≥ 2, Table S2). These landscapes, visualized via volcano plots and clustering heatmaps (Figure 2), demonstrate a profound and reproducible transcriptomic shift between HGSC and normal ovarian tissues.

### 2.2. Functional Convergence and Pathway Reprogramming

**Figure 3:**
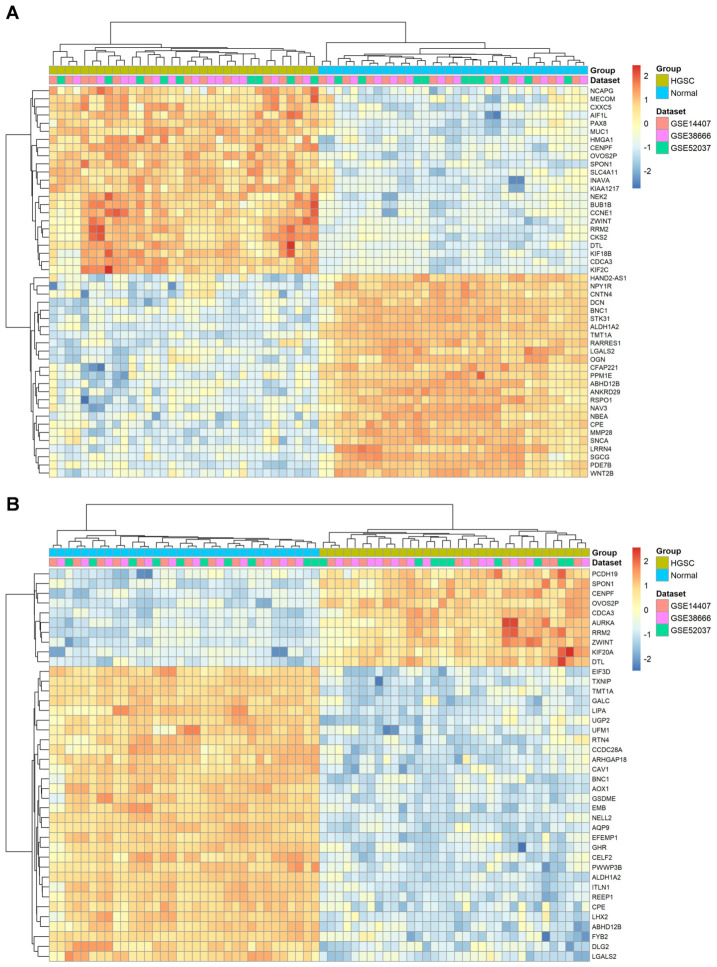
Cross-cohort heatmaps of meta-derived gene panels across HGSC vs. Normal samples. (**A**) Combined heatmap of z-scored expression levels for the top 48 meta-selected genes across all datasets, including 34 HGSC vs. 34 Normal samples. Genes were prioritized based on meta-analysis of differential expression (DEGs) using Benjamini–Hochberg FDR control (≤0.05) and ranked by statistical significance and effect size (direction indicated by meta logFC). Each column represents a single sample, and each row represents a gene. Expression values were standardized (z-scored) within each dataset before merging, enabling cross-platform visualization of relative expression patterns (blue = lower and red = higher relative expression). (**B**) Heatmap of z-scored expression levels for the top 40 panel genes across 34 HGSC vs. 34 Normal samples (*n* = 40 genes). This panel summarizes robust disease-associated transcriptional patterns, including key genes used in downstream predictive modeling. All expression values were gene-wise standardized within each dataset prior to integration.

**Figure 4:**
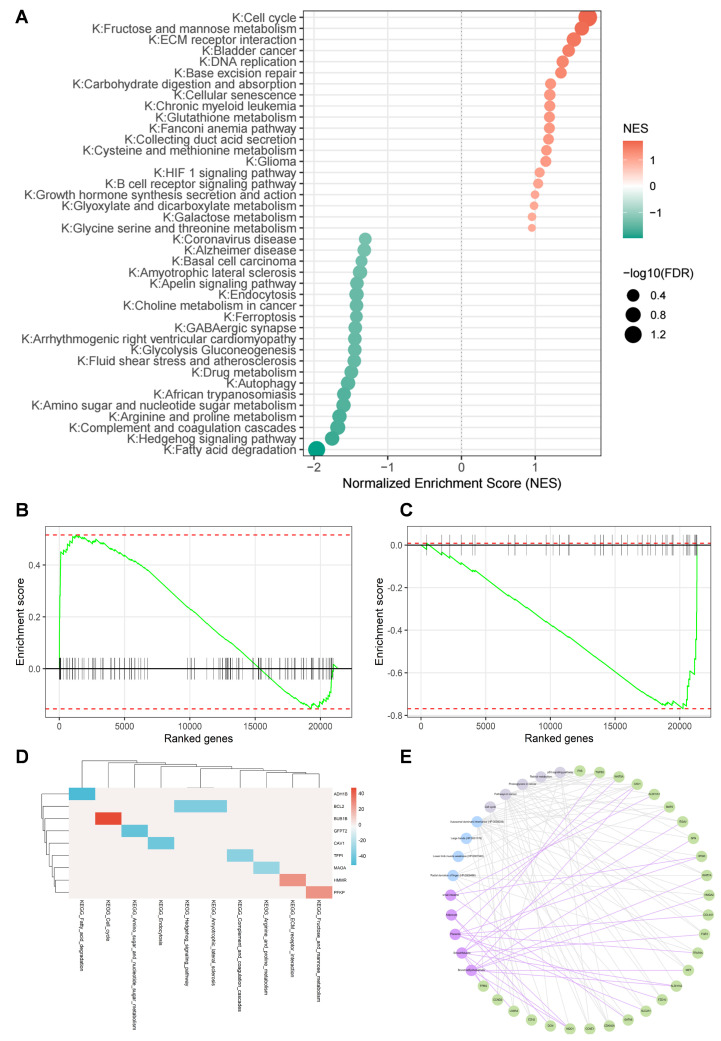
Functional enrichment analysis reveals pathway reprogramming in high-grade serous carcinoma (HGSC). (**A**) Gene set enrichment analysis (GSEA) was performed on a transcriptome-wide ranked gene list derived from the meta-analysis of discovery cohorts. This analysis identified significant pathway perturbations, with prominent up-regulated pathways including cell cycle and ECM–receptor interaction, and notably down-regulated pathways such as fatty acid degradation and Hedgehog signaling pathway. (**B**) Further highlighting key biological processes, GSEA underscored the cell cycle as a significantly enriched up-regulated pathway. (**C**) Conversely, fatty acid degradation emerged as a representative significantly down-regulated pathway, suggesting metabolic reprogramming in HGSC. (**D**) A heatmap illustrates the expression patterns of top-enriched genes across selected KEGG pathways, visually representing their coordinated activity. (**E**) Gene-pathway interaction networks were constructed using meta-analysis DEGs to elucidate relationships between key HGSC-associated genes and enriched pathways. The networks integrate annotations from KEGG, Human Phenotype Ontology (HPO), and the Human Gene Atlas. Enrichment scores were quantified for each pathway, and statistical significance was determined using adjusted *p*-values (FDR).

To move beyond single-gene lists, we integrated cohort-specific signals through a random-effects meta-analysis. A “meta-signature” of 48 top-ranked genes (Figure 3A) showed excellent discrimination and case–control separation across all platforms. To enhance visualization and resolve tissue-specific clustering, we filtered for the top 40 genes showing the most robust differential expression, effectively segregating the tumor and normal cohorts (Figure 3B). Functional enrichment (GSEA/KEGG) highlighted a coordinated upregulation of cell cycle and DNA replication machinery, while metabolic pathways, specifically fatty acid degradation, were systematically suppressed (Figure 4A–E).

### 2.3. Machine Learning and the 22-Gene Diagnostic Panel

**Figure 5:**
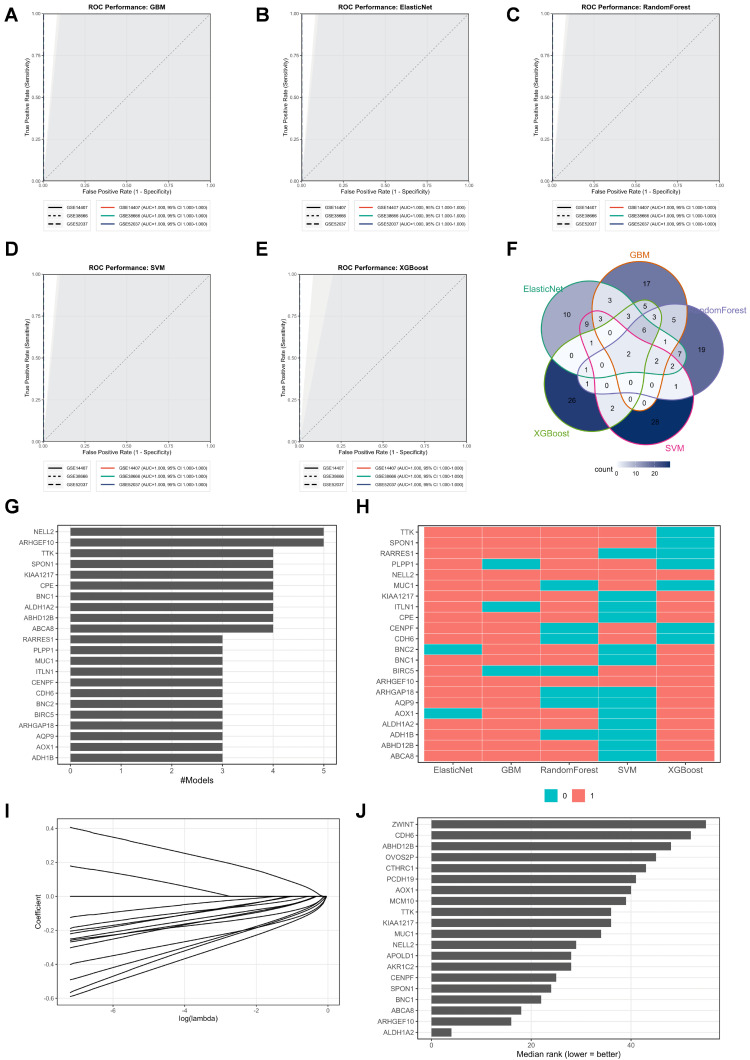
Machine learning performance and consensus feature discovery in high-grade serous carcinoma. (**A**–**E**) ROC curves showing the classification performance of five models under strict Leave-One-Dataset-Out validation. Each curve reflects the ability of a model to discriminate cases from controls in a held-out cohort. Overall, models achieved excellent performance, with the largest drop seen for XGBoost on GSE52037 (AUC ≈ 0.91). (**F**) Overlap among the top 50 features of each model, revealing a common core of genes consistently selected across classifiers. (**G**) Number of models selecting each gene (support count), highlighting the most stable and reproducible predictors. (**H**) Binary inclusion matrix showing which genes appear in the top 50 for each model (1 = selected). (**I**) ElasticNet/LASSO coefficient paths along the regularization trajectory, illustrating the stability of top-ranked genes under penalization. (**J**) Combined ranking across models (median rank), prioritizing genes that consistently emerge as key predictors.

**Figure 6:**
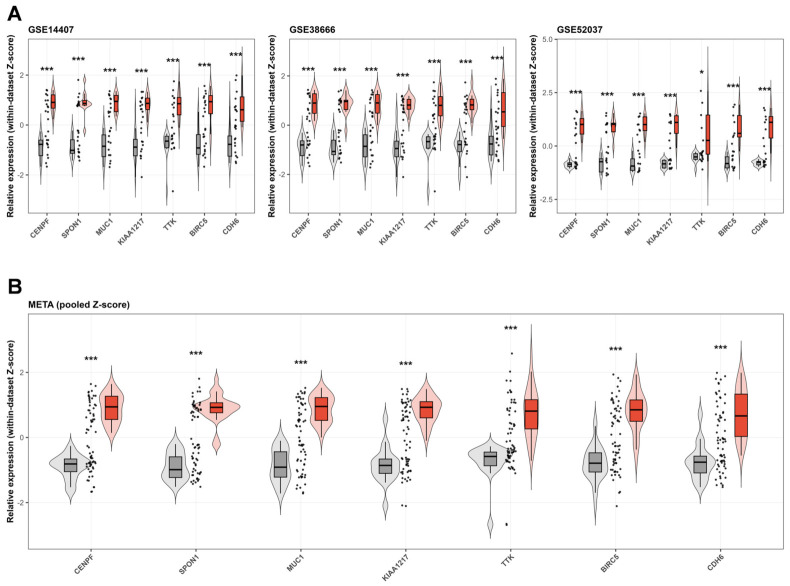
Final 7 up-regulated gene panel in the three GEO datasets vs. cross cohort. (**A**) Illustrates relative gene expression with Z-score in the vertical axis vs. 7 genes including *CENPF, SPON1, MUC1, KIAA1217, TTK, BIRC5* and *CDH6* in GSE14407, GSE38666, and GSE52037. (**B**) Meta-pooled and cross-cohorts gene panel shows consistency across cohorts. Normal samples are shown in gray; HGSC samples are colored warm orange for meta-up genes. HGSC vs. Normal differences were assessed per gene using a two-sided Wilcoxon rank-sum test on pooled Z-scores with Benjamini–Hochberg correction across 7 genes (* p < 0.05; *** *p* < 0.001).

**Figure 7:**
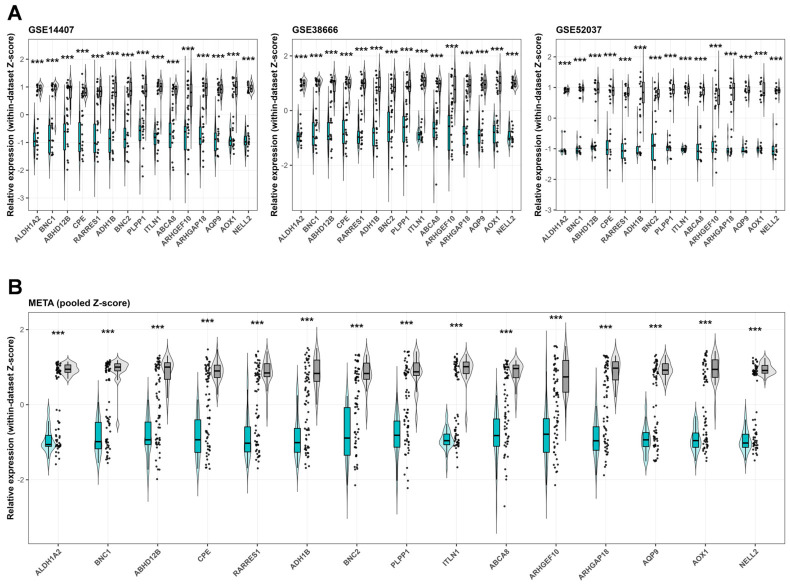
Final 15 down-regulated gene panel in the three GEO datasets vs. cross cohort. (**A**) Illustrates relative gene expression with Z-score in the vertical axis vs. 15 genes including *ALDH1A2, BNC1, ABHD12B, CPE, RARRES1, ADH1B, BNC2, PLPP1, ITLN1, ABCA8, ARHGEF10, ARHGAP18, AQP9, AOX1,* and *NELL2* in GSE14407, GSE38666, and GSE52037. (**B**) Meta-pooled and cross-cohorts gene panel shows consistency across cohorts. Normal samples are shown in gray; HGSC samples are colored turquoise for meta-down genes. HGSC vs. Normal differences were assessed per gene using a two-sided Wilcoxon rank-sum test on pooled Z-scores with Benjamini–Hochberg correction across 15 genes (*** *p* < 0.001).

**Figure 8:**
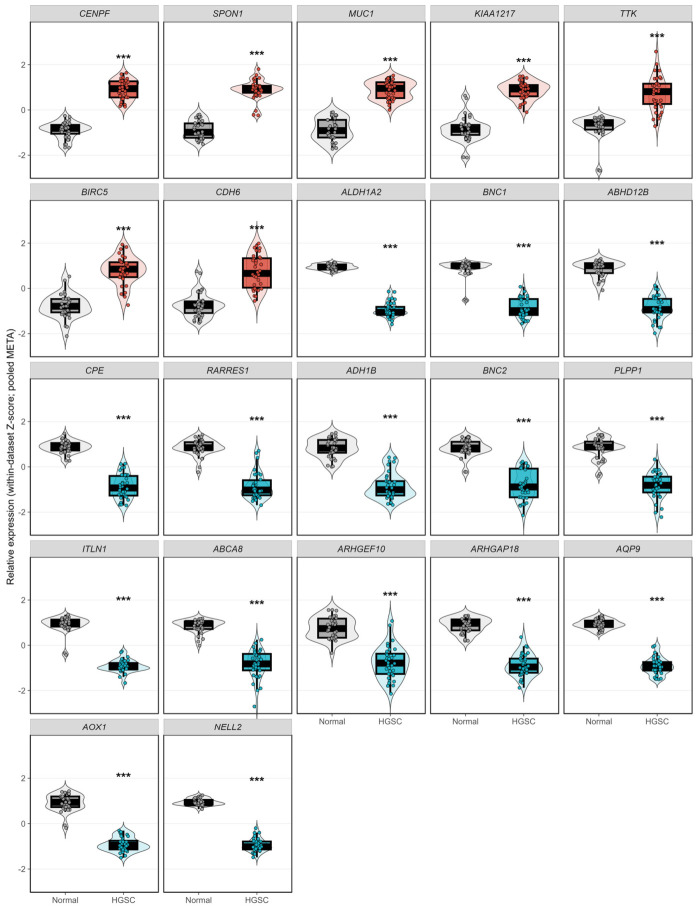
Meta-pooled expression visualization of the model-stable, meta-supported final 22-gene panel. Violin/box plots display pooled relative expression for final 22 genes (7 meta-up-regulated; 15 meta-down-regulated) across GSE14407, GSE38666, and GSE52037. The final 22-gene panel was defined upstream by requiring multi-model stability (TopK = 50 per model; retained if present in ≥3 classifiers) and meta-level significance (meta-FDR ≤ 0.05); the final 22 genes were prioritized by lower meta-FDR with *n*_models used as a secondary ranking criterion, and directionality was assigned by the sign of meta logFC. The expression values were converted to within-dataset gene-wise Z-scores and then pooled across datasets to form a single META distribution per gene. Normal samples are shown in gray; HGSC samples are colored warm orange for meta-up genes and turquoise for meta-down genes. HGSC vs. Normal differences were assessed per gene using a two-sided Wilcoxon rank-sum test on pooled Z-scores with Benjamini–Hochberg correction across 22 genes (*** *p* < 0.001).

**Figure 9:**
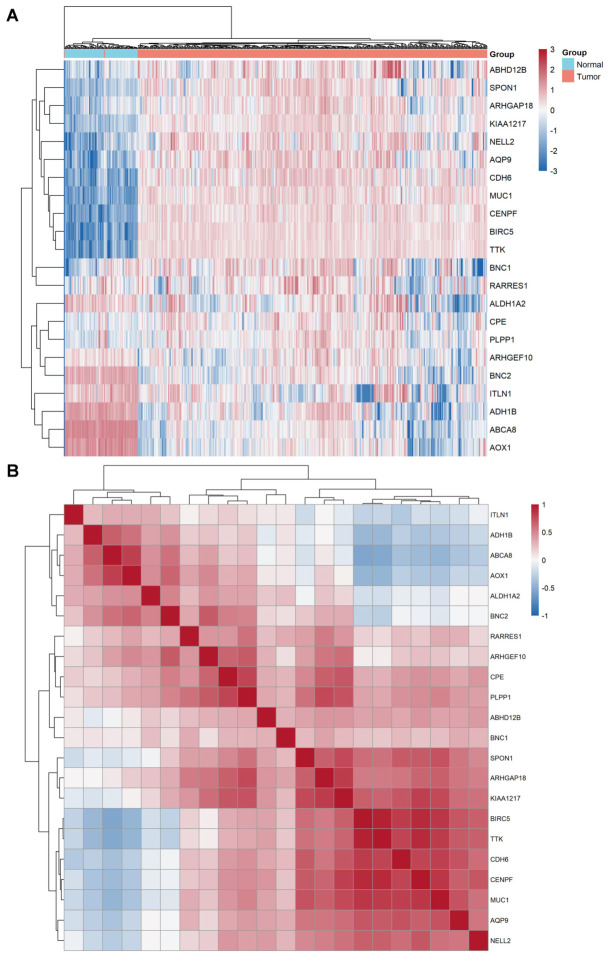
Validation of the 22-gene Transcriptomic Signature and Co-expression Landscape in TCGA-OV tumors (*n* = 427) versus GTEx normal ovaries (*n* = 88). (**A**) Differential Expression Profile in TCGA and GTEx Cohorts: The heatmap illustrates the expression landscape of the finalized 22-gene signature across HGSC and Normal ovarian tissues. To ensure cross-platform comparability, raw data from TCGA-OV (Tumor, *n* = 427) and GTEx (Normal Ovary, *n* = 88) integrated and batch-corrected using RSEM-normalized TPM values. Values are represented as Z-scores (row-scaled log2 (TPM + 0.001) to highlight relative up- and down-regulation. The hierarchical clustering (Euclidean distance, Complete linkage) reveals a distinct transcriptomic divergence between high-grade serous carcinoma (HGSC) and normal ovarian physiology, confirming the diagnostic robustness of the selected candidates. (**B**) Intragenic Co-expression Analysis: A correlation matrix (Heatmap) depicting the synergistic relationship between the 22 candidate genes. The matrix is constructed based on Spearman’s rank correlation coefficients (ρ) to capture both linear and non-linear associations within the tumor microenvironment. The color gradient (blue to red) indicates the strength of the correlation (−1 ≤ ρ ≤ 1). The emergence of tightly coupled gene clusters suggests potential functional modules involved in HGSC pathogenesis and risk stratification, further justifying the inclusion of these genes in the multi-gene prognostic model.

**Figure 10:**
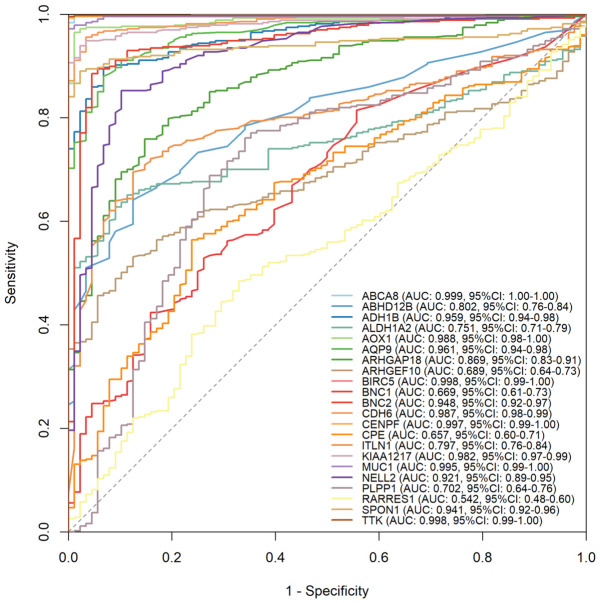
Diagnostic Performance and Robustness of the 22-gene Signature in TCGA-OV tumors (*n* = 427) versus GTEx normal ovaries (*n* = 88). Panel Multi-gene ROC Curve Analysis: The plot demonstrates the individual and collective diagnostic performance of the 22 candidate genes in discriminating high-grade serous carcinoma (HGSC) from normal ovarian tissue. Each colored curve represents a specific gene from the signature, validated across the integrated TCGA-OV and GTEx datasets. The diagonal dashed line indicates a performance equivalent to random chance (AUC = 0.50). High-performance trajectories across the majority of candidates (with AUC values ranging from min_AUC = 0.54 to max_AUC = 0.99) underscore the robust diagnostic power of this signature. The proximity of the curves to the upper-left quadrant confirms high sensitivity and specificity, validating these genes as reliable biomarkers for disease identification in large-scale independent cohorts.

Using a strict LODO (Leave-One-Dataset-Out) framework, we benchmarked five ML architectures. All models achieved excellent discrimination (AUC up to 1.00), though a slight performance dip in GSE52037 (AUC = 0.91) underscored the necessity of cross-cohort validation (Figure 5A–J, Table S3). By intersecting the top 50 features from each model, we identified a 22-gene consensus panel that maintained statistical significance (FDR < 0.05) and multi-model stability (Figure 6, Figure 7 and Figure 8). Clustering heatmaps, gene–gene correlation heatmaps, and ROC analyses of the 22-gene panel in TCGA-OV tumors (*n* = 427) and GTEx normal ovarian tissues (*n* = 88) are presented in Figure 9 and Figure 10.

### 2.4. Diagnostic and Prognostic Implications of ALDH1A2 and SPON1 Expression in HGSC

**Figure 11:**
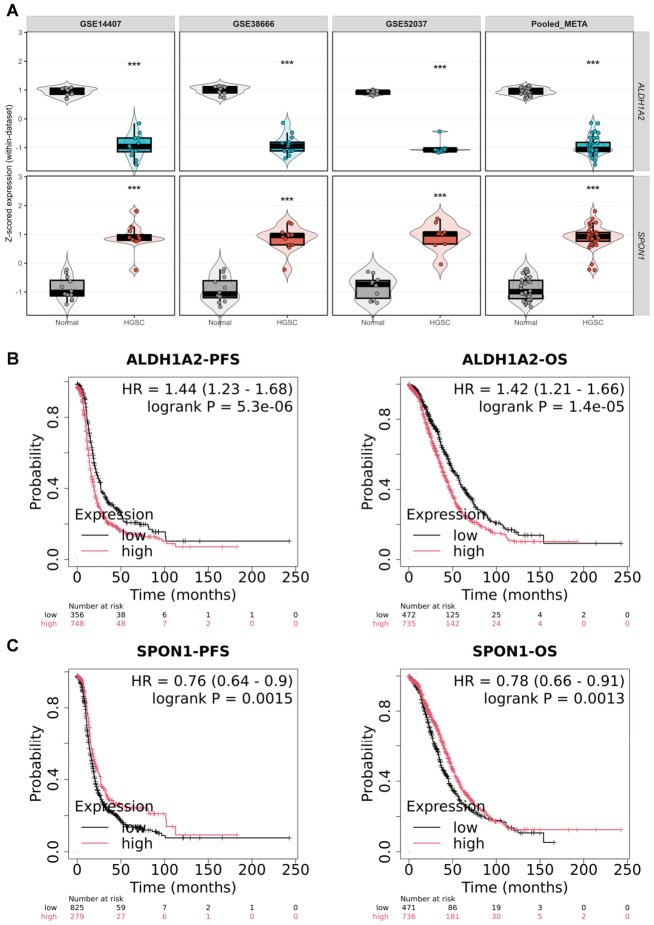
Validation of top consensus biomarkers (*ALDH1A2* and *SPON1*). (**A**) The core 2 candidate genes, *ALDH1A2* and *SPON1*, are the best ranked transcriptomic features consistently selected by all five machine-learning models, showing robust dysregulation and clear case–control separation across cohorts. Receiver Operating Characteristic (ROC) analysis evaluating the diagnostic efficacy of *ALDH1A2* and *SPON1* in HGSC patients. (**B**) Progression-free survival (PFS) and Overall survival (OS) stratified by *ALDH1A2* expression. (**C**) PFS and OS stratified by *SPON1* expression. Patients were dichotomized into high and low expression groups. Hazard ratios (HR), 95% confidence intervals, and log-rank *p* values are provided for each analysis. Note the opposing prognostic directions of the two genes, with high *ALDH1A2* indicating adverse outcomes and high *SPON1* indicating improved outcomes (significant value of log-rank *** *p* < 0.05).

To derive a clinically parsimonious model, we prioritized features with maximal cross-algorithm agreement. Only two genes, *ALDH1A2* and *SPON1* emerged as the “Core-2” candidates, consistently ranked at the top by all five models (Figure 11A). To evaluate the clinical significance of our candidate genes, we analyzed their association with patient survival using Kaplan–Meier estimates and univariate Cox proportional hazards regression. Our analysis revealed distinct prognostic patterns for *ALDH1A2* and *SPON1*. High *ALDH1A2* expression was significantly associated with inferior survival outcomes, with an HR of 1.44 (95% CI: 1.23–1.68, *p* = 5.3 × 10^−6^) for progression-free survival (PFS) and 1.42 (95% CI: 1.21–1.66, *p* = 1.4 × 10^−5^, Figure 11B) for overall survival (OS). Conversely, high *SPON1* expression demonstrated a protective effect, correlating with improved PFS (HR = 0.76, 95% CI: 0.64–0.90, *p* = 0.0015) and OS (HR = 0.78, 95% CI: 0.66–0.91, *p* = 0.0013, Figure 11C). These findings suggest that *ALDH1A2* and *SPON1* function as independent prognostic biomarkers with opposing clinical implications in HGSC.

### 2.5. Immuno-Transcriptomic Coupling

**Figure 12:**
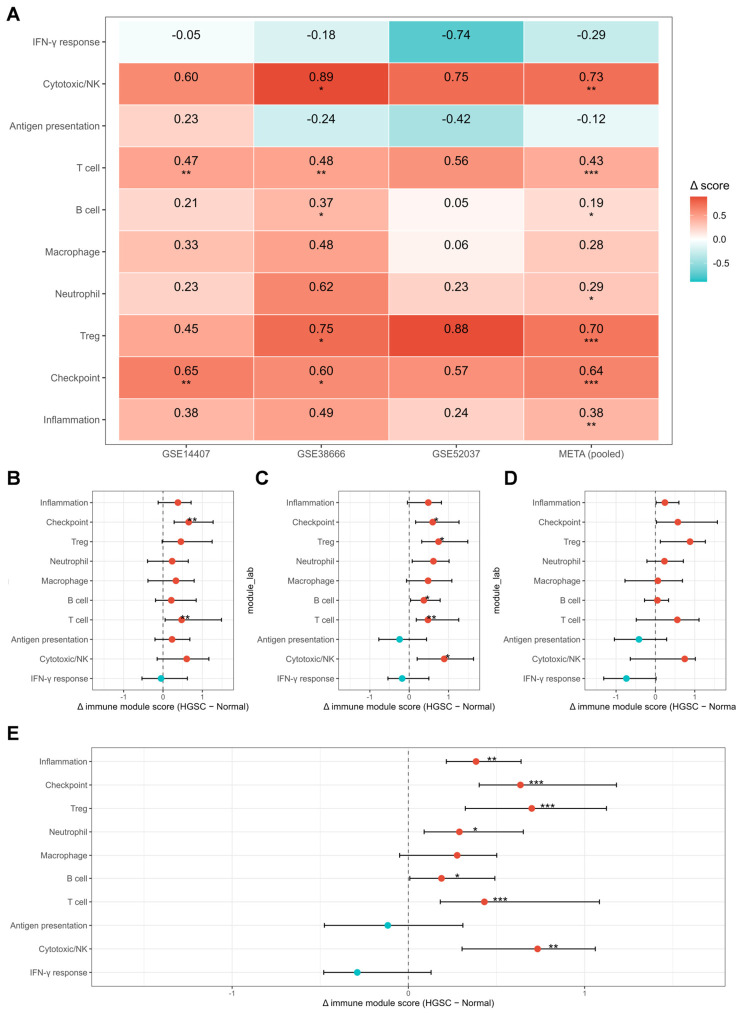
Immune module analysis reveals consistent immune alterations in HGSC. Cohort- and meta-level analyses demonstrate reproducible shifts in immune activity across HGSC samples. (**A**) Heatmap of median immune module changes (Δ = median HGSC median Normal) for ten predefined modules across GSE14407, GSE38666, GSE52037, and the pooled meta-analysis (“META”). Module scores were calculated per sample as the mean Z-scored expression of constituent marker genes within each cohort (modules included if ≥3 genes were available). (**B**–**D**) Forest/lollipop plots depicting Δ values for each cohort individually, with 95% confidence intervals. Points are colored by directionality (positive = higher in HGSC; negative = lower in HGSC). (**E**) Pooled (META) Δ estimates across cohorts after within-cohort standardization. Statistical significance is indicated by asterisks reflecting BH-FDR correction (* *p* < 0.05; ** *p* < 0.01; *** *p* < 0.001).

Using cohort-aware within-dataset standardization, group differences were summarized as Δ = median (HGSC)−median (Normal) for ten immune modules. In the pooled META analysis, seven modules showed significantly higher scores in HGSC after BH correction: Cytotoxic/NK (Δ = 0.7329, padj = 0.00613), T cell (Δ = 0.4313, padj = 2.90 × 10^−5^), B cell (Δ = 0.1879, padj = 0.01424), Neutrophil (Δ = 0.2894, padj = 0.01701), Treg (Δ = 0.6999, padj = 1.47 × 10^−5^), Checkpoint (Δ = 0.6357, padj = 1.07 × 10^−5^), and Inflammation (Δ = 0.3839, padj = 0.00747). In contrast, IFN-γ response (Δ = 0.0541, padj = 0.811) and Antigen presentation (Δ = 0.0392, padj = 0.893) were not significantly altered in the pooled analysis (Figure 12A).

Cohort-level results were directionally consistent but heterogeneous. In GSE14407, T cell (Δ = 0.4725, padj = 0.00557) and Checkpoint (Δ = 0.6528, padj = 0.00557) were significantly elevated, while Treg showed a positive but non-significant trend (Δ = 0.3984, padj = 0.0806). In GSE38666, broader immune activation was observed, with significant increases in Cytotoxic/NK (Δ = 0.8895, padj = 0.0270), T cell (Δ = 0.4757, padj = 0.00344), B cell (Δ = 0.3719, padj = 0.0270), Treg (Δ = 0.7456, padj = 0.0123), and Checkpoint (Δ = 0.5975, padj = 0.0123); Neutrophil and Inflammation were positive but did not reach significance after correction. GSE52037 showed positive shifts across several modules, including Treg (Δ = 0.8312) and Cytotoxic/NK (Δ = 0.7291), but none remained significant after multiple-testing correction (Figure 12B–D).

Importantly, all module deltas were positive across cohorts and in the pooled analysis, indicating consistently higher immune-module scores in HGSC than in normal tissue. The 95% confidence intervals further supported the robustness of the META-significant modules, including T cell (95% CI:0.1811 to 1.0838) and Checkpoint (95% CI: 0.4020 to 1.1807). Collectively, these results indicate that HGSC is characterized by a reproducible immune activation signature coupled to a prominent checkpoint/Treg axis, with cohort-specific variation in the breadth and statistical strength of the signal (Figure 12E).

### 2.6. Predicted HGSC Probability Aligns with Immune Modules

**Figure 13:**
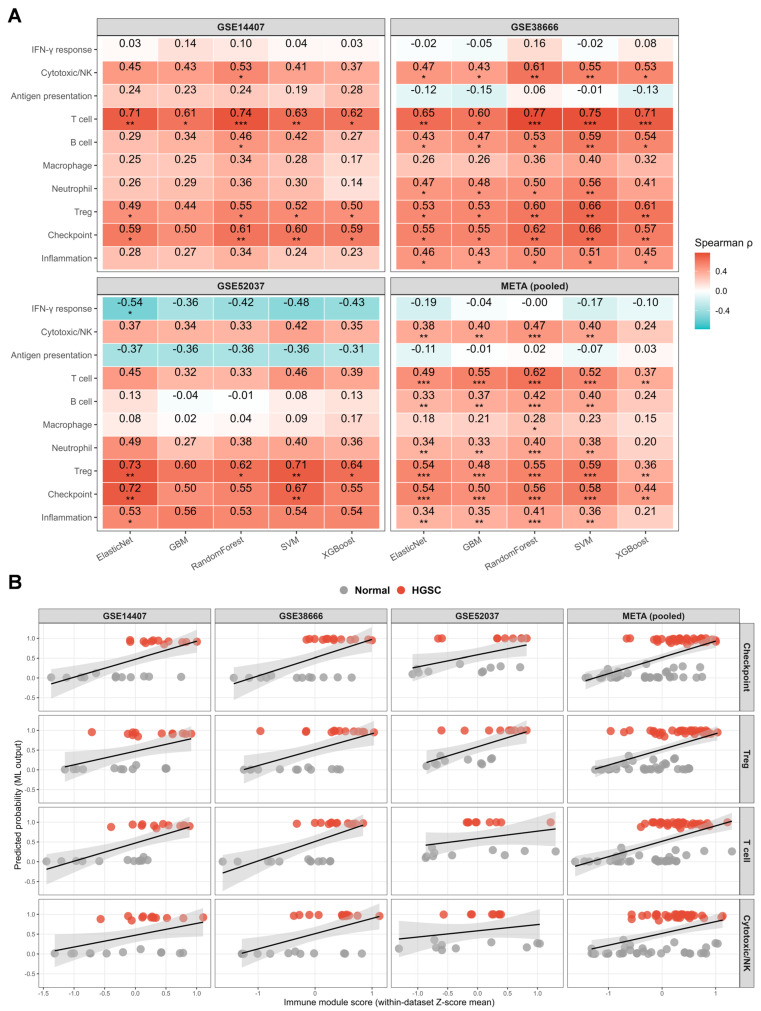
Machine-learning predicted HGSC probability is associated with immune module activity. (**A**) Heatmaps display Spearman correlation coefficients (ρ) between model-predicted HGSC probability and immune module scores for each cohort (GSE14407, GSE38666, GSE52037) and the pooled meta-analysis (“META”), across five ML models. Tile values represent ρ; asterisks indicate BH-FDR–adjusted significance within each dataset–model combination (* *p* < 0.05; ** *p* < 0.01; *** *p* < 0.001). (**B**) Scatter plots for the best-performing model (selected based on pooled AUC) illustrate the relationship between predicted HGSC probability and module scores for key immune modules (Checkpoint, Treg, T cell, Cytotoxic/NK) across cohorts and META. Points are colored by class (Normal vs. HGSC).

The strongest and most consistent associations in META were observed for lymphocyte and regulatory axes. Specifically, predicted probability showed moderate-to-strong positive correlations with T cell (Spearman ρ = 0.48–0.57 across models), Checkpoint (ρ = 0.47–0.61), Treg (ρ = 0.36–0.55), and Cytotoxic/NK (ρ = 0.28–0.40). More modest positive correlations were seen for Neutrophil (ρ = 0.21–0.34) and Macrophage (ρ = 0.11–0.22), whereas IFN-γ response was near-null to weakly negative (ρ = −0.11–0.02) and Antigen presentation remained weak overall (ρ = −0.02–0.12) in the pooled view. Cohort-wise patterns were directionally concordant but differed in magnitude. In GSE14407 and GSE38666, probability tracked T cell (ρ = 0.52–0.67 and 0.54–0.70, respectively) and Checkpoint (ρ = 0.44–0.61 and 0.48–0.63), alongside consistent positive coupling to Treg and Cytotoxic/NK (Figure 13A). In GSE52037, the probability signal showed particularly strong positive alignment with Treg (ρ = 0.43–0.73) and Checkpoint (ρ = 0.38–0.71), while exhibiting clear inverse correlations with IFN-γ response (ρ = −0.35 to −0.50) and Antigen presentation (ρ = −0.21 to −0.38) (Figure 13A), suggesting cohort-specific immune context that still maps onto the ML out-put. At the sample level, scatter panels for the best-performing model (by pooled AUC) confirmed a monotonic increase in predicted HGSC probability with increasing module scores for Checkpoint, Treg, T cell, and Cytotoxic/NK, with separation between Normal and HGSC samples consistent with the cohort-level associations (Figure 13B). Because correlation can be inflated by case–control separation, we additionally quantified within-class correlations (Normal and HGSC) and performed class-adjusted regression (prob ~ score + class) as sensitivity analyses, with full statistics provided in the corresponding output tables (Table S4).

## 3. Discussion

High-grade serous carcinoma (HGSC) remains the most lethal subtype of epithelial ovarian cancer, largely because most patients present with advanced-stage disease, extensive intraperitoneal spread, and ultimately develop recurrent, therapy-resistant tumors. In this setting, there is an urgent clinical need for biomarkers that are not only diagnostically robust, but also biologically informative and clinically actionable. Our findings support a translational framework in which cross-cohort transcriptomic stability can be leveraged to identify biomarkers with potential value in diagnosis, prognosis, and biological stratification of HGSC. In particular, the emergence of *ALDH1A2* and *SPON1* as the most consistently informative features suggests that these genes may capture complementary aspects of HGSC biology: one linked to tumor aggressiveness and adverse outcome, and the other associated with a comparatively more favorable disease course.

A central finding of the present study is that although the broader transcriptomic profile of HGSC is dominated by proliferation-associated pathways, the most clinically informative genes were not limited to canonical cell-cycle markers. This is important from a translational standpoint. Highly proliferative genes often perform well in distinguishing tumor from normal tissue, but they may have limited specificity for disease behavior because they reflect a general malignant state rather than biologically distinct vulnerabilities. By contrast, the consistent prioritization of *ALDH1A2* and *SPON1* across models suggests that these genes capture deeper organizational features of the HGSC phenotype, including cell-state plasticity, stromal interaction, and outcome-associated heterogeneity. This makes them more crucial as biomarkers for translational development than markers that are merely differentially expressed.

Among these two genes, *ALDH1A2* is arguably the more clinically provocative. In our analysis, *ALDH1A2* was reduced in tumor relative to normal tissue, yet higher expression within the HGSC cohort was associated with significantly worse progression-free and overall survival. This apparent paradox is biologically plausible and clinically meaningful. ALDH family enzymes have long been linked to stem-like phenotypes, detoxification capacity, aldehyde metabolism, and resistance to cytotoxic stress across multiple solid tumors, including ovarian cancer [11]. In ovarian malignancy, high ALDH activity has been repeatedly associated with tumor-initiating capacity, chemotherapy resistance, and enrichment of cancer stem-like populations [12]. Within that framework, the adverse prognostic effect of higher intratumoral *ALDH1A2* expression may indicate that residual expression marks a biologically privileged subpopulation with enhanced survival fitness under therapeutic pressure. In other words, although the tumor as a whole suppresses normal differentiation-associated programs, those tumors that preserve or re-activate ALDH-related metabolic circuitry may be better equipped to survive platinum exposure, repopulate after treatment, and recur early [13]. This interpretation is clinically relevant because it positions *ALDH1A2* not only as a prognostic biomarker, but also as a possible indicator of treatment persistence and chemoresistant cell states.

From a translational oncology perspective, this type of signal is more valuable than a simple “up-regulated oncogene” narrative. A gene that is globally down-regulated in cancer versus normal tissue but still stratifies poor-outcome patients within the tumor population may reflect a state biomarker, not just a disease marker. State biomarkers are particularly relevant in HGSC because clinically important variation often occurs within histologically similar tumors. If validated prospectively, *ALDH1A2* could therefore contribute to risk stratification after primary surgery or at the time of molecular triage, identifying patients at greater risk for early relapse even within the same histopathological class. Moreover, because aldehyde dehydrogenase biology intersects with drug tolerance and redox homeostasis, this signal may also be useful for prioritizing patients for intensified maintenance strategies or for trials targeting resistant cellular subpopulations [14].

In contrast, *SPON1* displayed the opposite clinical pattern, with higher expression associated with better survival outcomes. This is a particularly interesting observation because extracellular matrix and adhesion-associated genes are often interpreted simplistically as invasion-promoting [15]. However, the extracellular matrix is not uniformly pro-tumorigenic; rather, it encodes context-dependent cues that can either facilitate dissemination or maintain tissue organization. *SPON1*, a secreted extracellular matrix glycoprotein, may represent one such context-sensitive molecule [16]. Its favorable prognostic association in our data suggests that higher expression may reflect a less disordered, less plastic, or less aggressively remodeling tumor microenvironment. In practical clinical terms, *SPON1* may be identifying tumors with lower invasive potential or a more constrained stromal architecture, both of which could translate into slower progression and improved survival [17].

The diagnostic relevance of *SPON1* is also notable. Biomarkers that provide both strong discrimination between tumor and normal tissue and favorable prognostic annotation are relatively uncommon. Such dual-behavior markers may be especially useful in translational assay design because they can serve as anchors in reduced gene panels. In a clinical setting, a key panel containing a high-risk component *ALDH1A2* and a protective/stabilizing component *SPON1* may offer greater interpretability than larger signatures dominated by redundant proliferation genes. This is relevant for eventual implementation in qPCR-, NanoString-, or targeted RNA-based workflows, where assay simplicity, reproducibility, and interpretability are essential for clinical adoption.

Another clinically important finding in this study is the strong relationship between the HGSC transcriptional signal and the immune microenvironment [18]. We observed that higher predicted HGSC probability was positively associated with immune modules related to T cells, cytotoxic/NK cells, regulatory T cells, checkpoint signaling, and inflammatory programs. At first glance, the elevation of T cell and cytotoxic modules might appear favorable; however, the simultaneous enrichment of checkpoint and Treg-associated signatures suggests that HGSC is not simply an inflamed tumor, but rather an immunologically engaged yet functionally restrained one [19]. This interpretation is fully consistent with the broader clinical experience in ovarian cancer, where immune infiltration alone has not translated into uniformly successful responses to single-agent immune checkpoint blockade. In HGSC, the critical issue may not be the absence of immune recognition, but the presence of an exhausted or suppressed antitumor immune response [20].

This immune coupling has direct translational implications. If the molecular features that define HGSC also track with checkpoint-rich and Treg-enriched microenvironments, then transcriptomic classifiers such as the one developed here may have utility beyond diagnosis. They may help identify tumors with a “hot-but-suppressed” phenotype—tumors in which immune cells are present but ineffective. Such tumors are unlikely to be optimally treated by checkpoint blockade alone, but may be rational candidates for combination strategies, such as checkpoint inhibition with PARP inhibitors, anti-angiogenic therapy, or approaches aimed at Treg modulation and myeloid reprogramming. Therefore, the immune-associated findings of our study strengthen the argument that transcriptomic signatures can function as biologically meaningful stratification tools, not merely pattern-recognition devices.

## 4. Materials and Methods

### 4.1. Data Acquisition and Multi-Platform Integration

In the present study, we expanded and refined our previously published approach for cross-cohort gene identification [5]. Transcriptomic profiles were integrated from multiple repositories to ensure robust biomarker discovery and validation. Discovery Cohorts: Microarray datasets (GSE14407, GSE38666, and GSE52037) were retrieved from the Gene Expression Omnibus (GEO) via GEOquery [21]. Validation Cohort (RNA-Seq): Primary tumor samples from The Cancer Genome Atlas (TCGA-OV) were accessed via TCGAbiolinks (https://www.cancer.gov/ccg/research/genome-sequencing/tcga, 5 June 2026), and normal ovarian tissue profiles were obtained from the Genotype-Tissue Expression (GTEx) project, (https://www.genome.gov/Funded-Programs-Projects/Genotype-Tissue-Expression-Project) using the recount3 interface. All analyses were conducted in the R environment (v4.5.2, 31 October 2025).

### 4.2. Cross-Platform Preprocessing and Batch Mitigation

For microarray data, raw CEL files were processed using the Robust Multi-array Average (RMA) algorithm. For RNA-Seq (TCGA/GTEx), raw counts were harmonized and normalized using the Trimmed Mean of M-values (TMM) method via *edgeR*. To mitigate technical variability and platform-specific biases between TCGA and GTEx, the *ComBat* algorithm (*sva package*) was employed within an empirical Bayes framework. Data were then log2(CPM + 1) transformed to ensure homoscedasticity:

$$y_{ij}\;=\;\log2\left(\;\frac{C_{ij}\;+\;0.5}{N_{j}\;+\;10^{6}}\;\times 10^{6}\;+\;1\;\right)$$

where *C_ij_* represents the count for gene *i* in sample *j*, and *N_j_* denotes the library size. Probes and Ensembl IDs were mapped to HGNC symbols; for many-to-one mappings, the feature with the maximum Interquartile Range (IQR) was retained. Probe identifiers were mapped to HGNC gene symbols using the appropriate Bioconductor annotation packages (hgu133plus2.db for GPL570) [22]. When multiple probes mapped to the same gene, a single representative probe was selected using the maximum interquartile range (IQR) criterion to retain the most variable probe across samples.

### 4.3. Statistical Framework and Meta-Integration

Differential expression (DE) was modeled using empirical Bayes moderated t-statistics (limma). To quantify cross-study reproducibility, a Random-Effects Model (REML) was implemented via metafor to derive pooled effect sizes (meta_logFC). Statistical significance was strictly controlled using the Benjamini–Hochberg (BH) False Discovery Rate (FDR) [23]. A post hoc power analysis confirmed that the discovery scale (*n* = 34 per group) achieved 80% power (*α* = 0.05) to detect a Cohen’s d ≈ 0.68, ensuring sufficient sensitivity for moderate-to-large expression shifts.

$$d=\left(Z_{1-\frac{\alpha }{2}}+Z_{1-\beta }\right)\times \sqrt{\frac{1}{n1}+\frac{1}{n2}}$$

where *n*1 and *n*2 denote the sample sizes in the HGSC and normal groups, respectively. Using *n*1 = 34 and *n*2 = 34, with critical values *Z*1 − *α*/2 = 1.96 and *Z*1 − *β* = 0.84, the minimum detectable effect size is:

$$d\;=\;\left(1.96\;+\;0.84\right)\times \;\sqrt{\frac{1}{34}+\frac{1}{34}}\;\approx 0.68$$

### 4.4. Functional Enrichment Analysis of Meta-Ranked Transcriptomic Signatures

To functionally interpret the transcriptomic alterations identified in the discovery phase, we performed gene set enrichment analysis (GSEA) on a genome-wide ranked gene list derived from the random-effects meta-analysis across the three GEO cohorts. Briefly, differential expression was first estimated independently in each cohort using the limma R/Bioconductor package, after which cohort-specific effect sizes were integrated using metafor to obtain a unified meta-analysis statistic for each of the 21,355 shared genes, including directionality and significance. Genes were then ranked according to the signed meta-analysis signal and subjected to enrichment analysis using the fgsea package in R against Hallmark, Reactome, and KEGG gene sets retrieved from the Molecular Signatures Database via msigdbr. Enrichment results were summarized using normalized enrichment scores (NES) and Benjamini–Hochberg-adjusted *p*-values, and the most significant positively and negatively enriched pathways were visualized with ggplot2 and patchwork.

### 4.5. Machine Learning and Feature Stability

A strict Leave-One-Dataset-Out (LODO) framework was used to train five machine-learning models: GBM, Elastic Net [24], Random Forest [25], SVM [26], and XGBoost. Model hyperparameters (*α*, *λ*, and *mtry*) were tuned using 5-fold nested cross-validation restricted to the training data [27]. To improve robustness, a multi-model consensus rule was imposed: genes were retained only if they appeared among the Top-K ranked features in at least three models and remained statistically significant in the meta-analysis (FDR < 0.05). This strategy identified a final 22-gene signature, which was subsequently reduced to a core two-gene panel (*ALDH1A2* and *SPON1*) based on Top-K feature overlap.

### 4.6. Diagnostic and Prognostic Validation

The diagnostic accuracy was quantified using Receiver Operating Characteristic (ROC) analysis (*pROC* package), with the Area Under the Curve (AUC) and DeLong 95% confidence intervals [28]. For prognostic utility, a Multivariate Cox Proportional Hazards Regression model was utilized. The patient-specific Risk Score was calculated as:

$$Risk\;Score=\sum_{i=1}^{n}\left(\beta i\times Expi\right)$$

where *βi* is the Cox regression coefficient and *Expi* is the normalized expression. Patients were stratified by the median risk score, and survival distributions were compared using Kaplan–Meier curves and log-rank tests (*survival* and *survminer* packages).

### 4.7. Tumor Microenvironment (TME) and Correlation Analysis

Immune infiltration was quantified using ssGSEA-lite, where module scores were calculated as the mean Z-score of constituent genes within each dataset. The immune modules were derived from curated gene signatures representing 10 key immune populations and processes (IFN-γ response, Cytotoxic/NK, and Checkpoints). To ensure statistical robustness, 95% confidence intervals (CIs) for the scores were estimated using non-parametric bootstrap resampling with replacement (300 iterations). To link the machine learning (ML) decision signals to TME biology, Spearman’s rank correlation (ρ) was computed between the ML-predicted probabilities and the immune module scores. To eliminate potential class-driven inflation, a class-adjusted linear regression was performed, and only associations independent of the case–control status were reported as biologically significant [29]. *p*-values were adjusted for multiple testing using the Benjamini–Hochberg (BH) FDR method.

### 4.8. Software and Statistical Analysis

All analyses were executed in the R environment (v4.5.2). Data acquisition was standardized using *TCGAbiolinks*, *recount3*, and *GEOquery*. High-throughput preprocessing, including TMM normalization and *ComBat*-based batch correction, was performed via *edgeR* and *sva*. Machine learning and diagnostic evaluations (ROC/AUC) utilized the *pROC* and *caret* ecosystems, while survival dynamics were modeled using *survival* and *survminer*. Comprehensive data wrangling and visualization were streamlined through the *tidyverse* suite, *data.table*, and *ggplot2*, ensuring publication standard. Statistical significance was rigorously defined at *p* < 0.05 using BH-FDR correction where applicable.

## 5. Conclusions

We developed a rigorous cross-cohort transcriptomic and machine learning framework that identified a reproducible 22-gene HGSC signature and a parsimonious two-gene core consisting of *ALDH1A2* and *SPON1*. External validation supported the robustness of this signature, while survival analyses indicated distinct and opposite prognostic associations for the two core biomarkers. Furthermore, immune-module profiling showed that the diagnostic transcriptomic signal is closely linked to an immune-active yet immunoregulatory tumor microenvironment. Together, these findings support the translational potential of *ALDH1A2* and *SPON1* for molecular classification, prognostic stratification, and biologically informed characterization of HGSC.

## Data Availability

All analyses were performed using a scripted R pipeline archived on GitHub (https://github.com/heidarzadehroozbeh-cmyk/ML-Prognostic_Biomarkers-for-Serous-OvarianCancer, 15 February 2026) and Zenodo (https://zenodo.org/records/20262520, 15 February 2026). The provided code documents the complete workflow from raw data acquisition and meta-analysis to machine learning benchmarking. The datasets analyzed in this study are publicly available and can be accessed through the following repositories: Gene Expression Omnibus (GEO) for GSE14407 (https://www.ncbi.nlm.nih.gov/geo/query/acc.cgi?acc=GSE14407, 15 February 2026), GSE38666, (https://www.ncbi.nlm.nih.gov/geo/query/acc.cgi?acc=GSE38666, 15 February 2026), and GSE52037 (https://www.ncbi.nlm.nih.gov/geo/query/acc.cgi?acc=GSE52037, 15 February 2026), and the GDC Data Portal for the TCGA-OV cohort (https://portal.gdc.cancer.gov/, 5 June 2026). Additionally, normal ovarian tissue expression profiles were retrieved from the GTEx Portal (https://gtexportal.org/, 5 June 2026). All processed data matrices, including the 22-gene signature expression profiles and the integrated risk score models, are available from the corresponding author upon reasonable request.

## Supplementary Materials

The following supporting information can be downloaded at: https://www.mdpi.com/article/10.3390/ijms27146263/s1.

## Abbreviations

The following abbreviations are used in this manuscript:

HGSC	High-Grade Serous Carcinoma
TCGA	The Cancer Genome Atlas Program
GTEx	Genotype-Tissue Expression
GEO	Gene Expression Omnibus
DEGs	Differential Expression
ML	Machine Learning
LODO	Leave-One-Dataset-Out
REML	Random-Effects Model
GSEA	Gene Set Enrichment Analysis
NES	Normalized Enrichment Scores
TME	Tumor Microenvironment

## References

[1] Bray F., Laversanne M., Sung H., Ferlay J., Siegel R.L., Soerjomataram I., Jemal A. (2024). Global cancer statistics 2022: GLOBOCAN estimates of incidence and mortality worldwide for 36 cancers in 185 countries. CA Cancer J. Clin..

[2] Kuroki L., Guntupalli S.R. (2020). Treatment of epithelial ovarian cancer. BMJ.

[3] Menon U., Gentry-Maharaj A., Burnell M., Singh N., Ryan A., Karpinskyj C., Carlino G., Taylor J., Massingham S.K., Raikou M. (2021). Ovarian cancer population screening and mortality after long-term follow-up in the UK Collaborative Trial of Ovarian Cancer Screening (UKCTOCS): A randomised controlled trial. Lancet.

[4] Anderson N.M., Simon M.C. (2020). The tumor microenvironment. Curr. Biol..

[5] Heidarzadehpilehrood R., Ling K.-H., Abdul Hamid H. (2026). Integrative transcriptomic analysis of WNT/TGFβ-driven EMT pathways and drug-gene interaction networks in epithelial ovarian cancer. Adv. Cancer Biol.—Metastasis.

[6] Tran K.A., Kondrashova O., Bradley A., Williams E.D., Pearson J.V., Waddell N. (2021). Deep learning in cancer diagnosis, prognosis and treatment selection. Genome Med..

[7] Zou H., Hastie T. (2005). Regularization and variable selection via the elastic net. J. R. Stat. Soc. Ser. B Stat. Methodol..

[8] Kavzoglu T., Teke A. (2022). Predictive Performances of Ensemble Machine Learning Algorithms in Landslide Susceptibility Mapping Using Random Forest, Extreme Gradient Boosting (XGBoost) and Natural Gradient Boosting (NGBoost). Arab. J. Sci. Eng..

[9] Montesinos López O.A., Montesinos López A., Crossa J. (2022). Support Vector Machines and Support Vector Regression. Multivariate Statistical Machine Learning Methods for Genomic Prediction.

[10] de Hond A.A.H., Steyerberg E.W., van Calster B. (2022). Interpreting area under the receiver operating characteristic curve. Lancet Digit. Health.

[11] Jackson B., Brocker C., Thompson D.C., Black W., Vasiliou K., Nebert D.W., Vasiliou V. (2011). Update on the aldehyde dehydrogenase gene (ALDH) superfamily. Hum. Genom..

[12] Kim D., Choi B.-H., Ryoo I.-G., Kwak M.-K. (2018). High NRF2 level mediates cancer stem cell-like properties of aldehyde dehydrogenase (ALDH)-high ovarian cancer cells: Inhibitory role of all-trans retinoic acid in ALDH/NRF2 signaling. Cell Death Dis..

[13] Muralikrishnan V., Fang F., Given T.C., Podicheti R., Chtcherbinine M., Metcalfe T.X., Sriramkumar S., O’hagan H.M., Hurley T.D., Nephew K.P. (2022). A Novel ALDH1A1 Inhibitor Blocks Platinum-Induced Senescence and Stemness in Ovarian Cancer. Cancers.

[14] Zhang Z., Tan Y., Huang C., Wei X. (2023). Redox signaling in drug-tolerant persister cells as an emerging therapeutic target. EBioMedicine.

[15] Miyakawa R., Kobayashi M., Sugimoto K., Endo Y., Kojima M., Kobayashi Y., Furukawa S., Honda T., Watanabe T., Asano S. (2023). SPON1 is an independent prognostic biomarker for ovarian cancer. J. Ovarian Res..

[16] Whately K.M., Sengottuvel N., Edatt L., Srivastava S., Woods A.T., Tsai Y.S., Porrello A., Zimmerman M.P., Chack A.C., Jefferys S.R. (2024). Spon1+ inflammatory monocytes promote collagen remodeling and lung cancer metastasis through lipoprotein receptor 8 signaling. JCI Insight.

[17] Nagasawa S., Ikeda K., Shintani D., Yang C., Takeda S., Hasegawa K., Horie K., Inoue S. (2022). Identification of a Novel Oncogenic Fusion Gene SPON1-TRIM29 in Clinical Ovarian Cancer That Promotes Cell and Tumor Growth and Enhances Chemoresistance in A2780 Cells. Int. J. Mol. Sci..

[18] Stur E., Peng F., Teng P.-N., Bayraktar E., Hu M., Corvigno S., Brown D.J., Lee S., Moore K.N., Bateman N.W. (2025). The dynamic immune behavior of primary and metastatic ovarian carcinoma. npj Precis. Oncol..

[19] Miceska S., Grašič Kuhar C., Frković Grazio S., Škof E., Krishnamoorthy P., Khabele D., Kloboves Prevodnik V. (2025). Association of Tumor-Infiltrating Lymphocytes and Inflammation Status with Survival Outcome in Patients with High-Grade Serous Ovarian Carcinoma. Cancers.

[20] Ratka Z., Gamian A., Woźniak M. (2026). Limited Clinical Benefit of Immune Checkpoint Inhibition in Ovarian Cancer with Opportunities in Selected Subtypes. Int. J. Mol. Sci..

[21] Sean D., Meltzer P.S. (2007). GEOquery: A bridge between the Gene Expression Omnibus (GEO) and BioConductor. Bioinformatics.

[22] Ritchie M.E., Phipson B., Wu D., Hu Y., Law C.W., Shi W., Smyth G.K. (2015). limma powers differential expression analyses for RNA-sequencing and microarray studies. Nucleic Acids Res..

[23] Benjamini Y., Hochberg Y. (1995). Controlling the False Discovery Rate: A Practical and Powerful Approach to Multiple Testing. J. R. Stat. Soc. Ser. B Methodol..

[24] Chen T., Guestrin C. (2016). XGBoost: A Scalable Tree Boosting System. Proceedings of the ACM SIGKDD International Conference on Knowledge Discovery and Data Mining.

[25] Hänzelmann S., Castelo R., Guinney J. (2013). GSVA: Gene set variation analysis for microarray and RNA-seq data. BMC Bioinform..

[26] Hesterberg T. (2011). Bootstrap. Wiley Interdiscip. Rev. Comput. Stat..

[27] Bindea G., Mlecnik B., Tosolini M., Kirilovsky A., Waldner M., Obenauf A.C., Angell H., Fredriksen T., Lafontaine L., Berger A. (2013). Spatiotemporal dynamics of intratumoral immune cells reveal the immune landscape in human cancer. Immunity.

[28] Friedman J.H. (2001). Greedy function approximation: A gradient boosting machine. Ann. Stat..

[29] Rooney M.S., Shukla S.A., Wu C.J., Getz G., Hacohen N. (2015). Molecular and genetic properties of tumors associated with local immune cytolytic activity. Cell.

