# Supplementary material for: Machine Learning-Guided Multi-Cohort Transcriptomic Profiling Identifies SPON1 and ALDH1A2 as Diagnostic and Prognostic Biomarkers Linked to the Immune Microenvironment in High-Grade Serous Carcinoma
Source: Int J Mol Sci. 2026 Jul 14;27(14):6263. doi: 10.3390/ijms27146263 (PMC13412053; doi:10.3390/ijms27146263)
Supplement: Supplementary file 1 [file ijms-27-06263-s001.zip › Figure S1 & S2.pptx]

## Slide 1
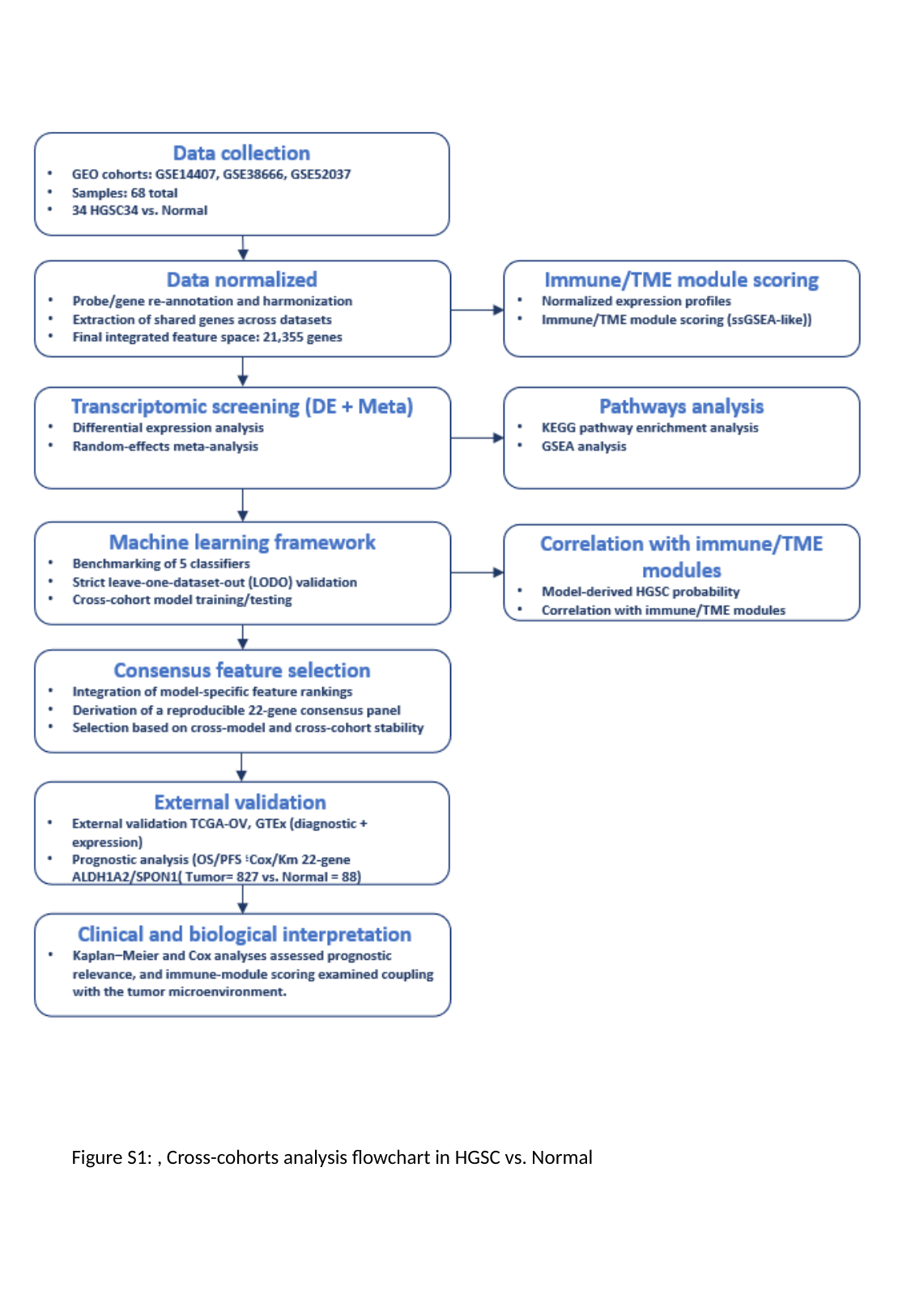

Figure S1: , Cross-cohorts analysis flowchart in HGSC vs. Normal

## Slide 2
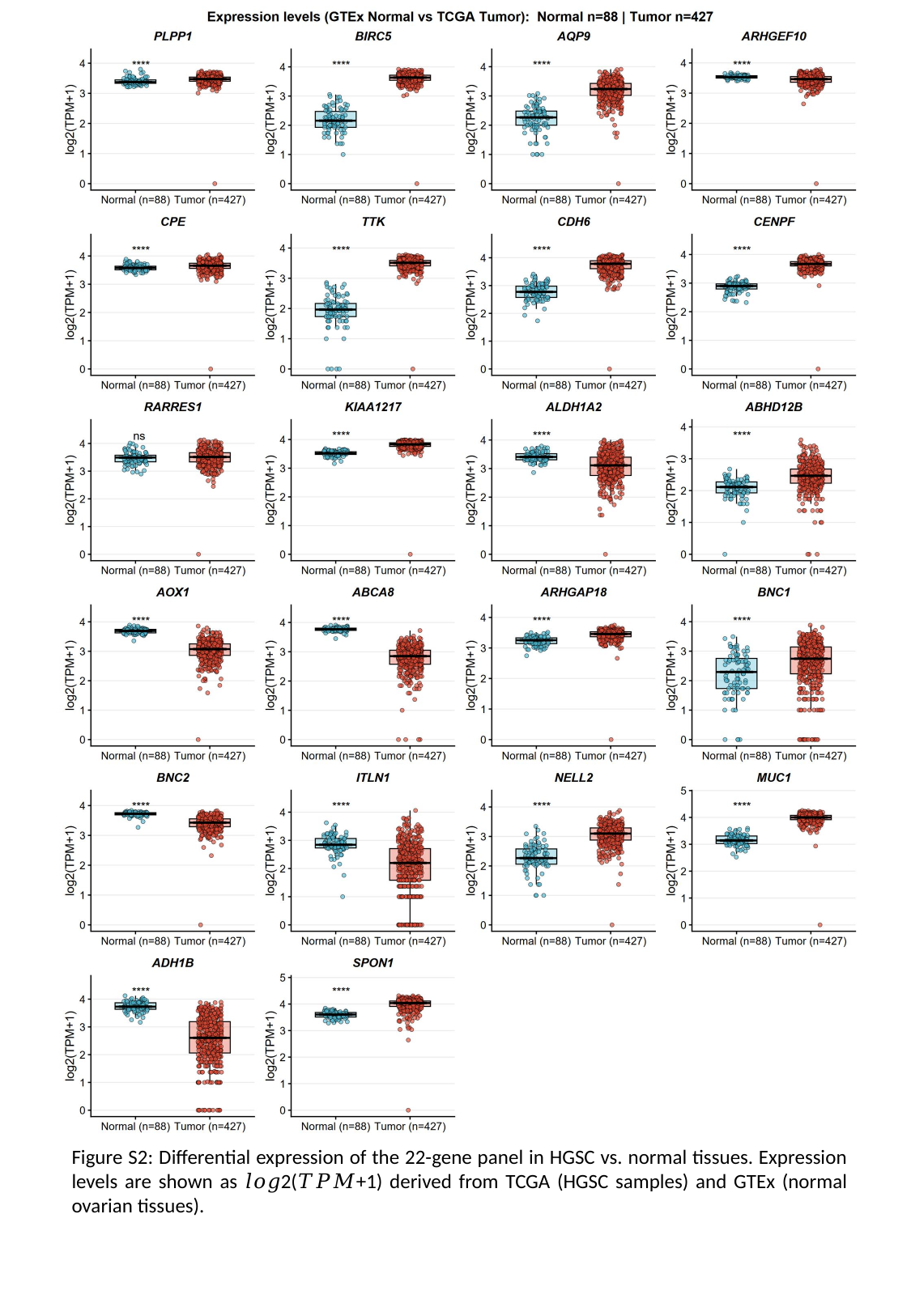

Figure S2: Differential expression of the 22-gene panel in HGSC vs. normal tissues. Expression levels are shown as 𝑙𝑜𝑔2(𝑇𝑃𝑀+1) derived from TCGA (HGSC samples) and GTEx (normal ovarian tissues).
